# Long-term consequences of chronic fluoxetine exposure on the expression of myelination-related genes in the rat hippocampus

**DOI:** 10.1038/tp.2015.145

**Published:** 2015-09-22

**Authors:** Y Kroeze, D Peeters, F Boulle, D L A van den Hove, H van Bokhoven, H Zhou, J R Homberg

**Affiliations:** 1 Department of Cognitive Neuroscience, Centre for Neuroscience, Donders Institute for Brain, Cognition, and Behaviour, Radboud University Medical Centre, Nijmegen, The Netherlands; 2 Department of Human Genetics, Centre for Neuroscience, Donders Institute for Brain, Cognition, and Behaviour, Radboud University Medical Centre, Nijmegen, The Netherlands; 3 School for Mental Health and Neuroscience, Maastricht University, European Graduate School of Neuroscience, Maastricht, The Netherlands; 4 Department of Molecular Developmental Biology, Faculty of Science, Radboud institute for Molecular Life Sciences, Radboud University Nijmegen Medical Centre, Nijmegen, The Netherlands

## Abstract

The selective serotonin reuptake inhibitor (SSRI) fluoxetine is widely prescribed for the treatment of symptoms related to a variety of psychiatric disorders. After chronic SSRI treatment, some symptoms remediate on the long term, but the underlying mechanisms are not yet well understood. Here we studied the long-term consequences (40 days after treatment) of chronic fluoxetine exposure on genome-wide gene expression. During the treatment period, we measured body weight; and 1 week after treatment, cessation behavior in an SSRI-sensitive anxiety test was assessed. Gene expression was assessed in hippocampal tissue of adult rats using transcriptome analysis and several differentially expressed genes were validated in independent samples. Gene ontology analysis showed that upregulated genes induced by chronic fluoxetine exposure were significantly enriched for genes involved in myelination. We also investigated the expression of myelination-related genes in adult rats exposed to fluoxetine at early life and found two myelination-related genes (*Transferrin (Tf)* and *Ciliary neurotrophic factor (Cntf)*) that were downregulated by chronic fluoxetine exposure. *Cntf*, a neurotrophic factor involved in myelination, showed regulation in opposite direction in the adult versus neonatally fluoxetine-exposed groups. Expression of myelination-related genes correlated negatively with anxiety-like behavior in both adult and neonatally fluoxetine-exposed rats. In conclusion, our data reveal that chronic fluoxetine exposure causes on the long-term changes in expression of genes involved in myelination, a process that shapes brain connectivity and contributes to symptoms of psychiatric disorders.

## Introduction

Selective serotonin reuptake inhibitors (SSRIs) are widely prescribed for the treatment of a variety of psychiatric disorders, such as depression,^1, 2^ bipolar affective disorder,^3^ anxiety-related disorders (obsessive compulsive disorder, post-traumatic stress disorder),^4, 5^ aggression^6^ and autism spectrum disorder.^7, 8^ All these disorders have symptoms related to, among others, negative affectivity, which might be the symptom responsive to SSRI treatment. Clinical studies have shown that there are many side effects upon chronic administration of SSRIs, like sexual dysfunction,^9^ suppression of rapid eye movement sleep,^10^ nausea,^11, 12^ decreased appetite^13^ and deterioration of symptoms (for example, aggression),^14, 15^ indicating that optimization of chronic treatment of affective disorders is needed. In addition, some patients remain free of symptoms after discontinuation of SSRI treatment, whereas in others the symptoms reoccur.^16, 17^ Studies in generalized social anxiety disorder patients showed that within 24 weeks after discontinuation of SSRI treatment 40–50% of the patients (receiving placebo after stopping SSRI) relapsed.^18, 19^ For major depressive disorder, the cumulative probability of suffering a recurrence of major depression within 2 years after discontinuation of SSRIs was 60% for people aged 60 years or older.^20^ Hence, the effects of SSRIs are not always sufficient to warrant long-term remission. To further improve the efficacy of SSRIs, there is an urgent need to unravel the mechanisms contributing to the long-term effects of chronic SSRI exposure.

The primary target of SSRIs is the serotonin transporter (5-HTT); its blockade by SSRIs leads to increases in extracellular serotonin (5-HT) levels. According to classic literature, chronic rises in 5-HT levels would contribute to the therapeutic effects of SSRIs,^21^ acting through 5-HT receptors. For example, antagonism of 5-HT_2C_ and 5-HT_7_ results in significantly increased therapeutic effects of SSRIs^22, 23, 24, 25^ and SSRI/5-HT_1A_ antagonist co-administration leads to increased extracellular 5-HT levels and enhanced antidepressant responses.^26, 27^ However, these and other 5-HT receptors are also implicated in the side effects of SSRIs, like sexual dysfunction (5-HT_1A_),^28^ sleep disturbances (5-HT_7_,^29^ 5-HT_1A_^30^), nausea (5-HT_3B_^31^) and decreased appetite (5-HT_2C,_^32^ 5-HT_1B_^33^). Although 5-HT receptors have a key role in the short-term effects of chronic SSRI exposure, it is likely that pathways downstream of the receptors are important for the long-term effects of chronic SSRI exposure.

Recently, several gene expression studies in rodents have shown that SSRI exposure can affect a wide variety of pathways in addition to the serotonergic system. It has been shown that SSRI treatment during adulthood alters gene expression levels of hypothalamic hormones, neurotrophic factors, inflammatory factors and components of non-serotonergic neurotransmitter systems.^34, 35, 36^ Especially the inflammatory factors might have a central role in mediating the effects of SSRIs, because downregulation of proinflammatory cytokines can inhibit HPA axis function (facilitating stress reduction), enhance 5-HT and dopamine synthesis and inhibit 5-HT and dopamine reuptake.^34^ Yet, these findings reflect short-term effects (24 h after the last treatment) of chronic SSRI exposure. The long-term consequences of SSRI exposure on gene expression are so far not well understood and might provide additional information about the long-term adaptations that contribute to the remediation of disease symptoms after stopping medication.

Studies addressing the long-term consequences of perinatal SSRI exposure may provide hints regarding potential mechanisms by which SSRIs exert their long-term effects. In humans and rodents, there is evidence that perinatal SSRI exposure increases the likelihood of symptoms related to autism^37, 38, 39, 40, 41^ in the offspring. This seemingly contrasts the use of SSRIs in the treatment of autism during adulthood. In addition, in rodents perinatally exposed to SSRIs there is evidence for ‘paradoxical' anxiety- and depression-like symptoms at adulthood.^42, 43, 44^ Because adult and perinatal SSRI exposure is associated with comparable effects on the serotonergic system, like increases in 5-HT levels, reductions in 5-HT transporter expression^45, 46^ and desensitization of 5-HT_1A_ receptors,^47, 48^ the ‘paradoxical' outcomes of perinatal SSRI exposure cannot be explained by 5-HT levels (alone). Critically, during development, 5-HT not only acts as a neurotransmitter, but also as a neurotrophic factor. Specifically, during early brain development, 5-HT steers neurodevelopmental processes like neuronal outgrowth and migration processes.^49, 50, 51^ Studies have shown that 5-HT affects embryonic interneuron migration^51^ and also affects organization of axonal projections of excitatory spiny stellate and pyramidal cells in the barrel cortex.^52^ These data show that 5-HT affects the outgrowth and migration of non-serotonergic neurons. As the brain is highly plastic during early development, rises in 5-HT levels induced by perinatal SSRI exposure can have outcomes that are substantially different from adult SSRI exposure. Nonetheless, studies focusing on early-life SSRI exposure could lead to potential targets of the long-term chronic SSRI exposure during adulthood. For example, SSRI exposure during brain development can disturb myelin sheath formation at adulthood^40^ and there is also evidence that SSRI treatment at adulthood can cause changes in white matter microstructure, which consists mainly of myelinated axons.^53^ Furthermore, both adult and developmental SSRI exposure can affect hippocampal neurogenesis at adulthood.^54, 55^

To more concretely elucidate the long-term effects of chronic SSRI exposure during adulthood, we investigated the long-term consequences of chronic fluoxetine (12 mg kg^−1^) versus vehicle treatment during adulthood (postnatal day (PND) 67–88) on gene expression in the hippocampus, a brain region that is highly responsive to SSRIs^55, 56^ and implicated in psychiatric disorders characterized by affective changes like anxiety,^57, 58^ bipolar affective disorder,^59^ aggression^60^ and depression.^61^ It has, for example, been shown that the hippocampus is directly involved in the mediation of unconditioned anxiety-related responses in animals.^57^ We measured body weight during treatment, as fluoxetine is known to exert anorectic effects.^62, 63^ In addition, we measured anxiety-like behavior in the novelty-suppressed feeding test (NSFT), which is highly sensitive to SSRIs.^42, 43, 64, 65, 66^ We studied genome-wide gene expression using transcriptome analysis (RNA-seq) in the hippocampal tissue of fluoxetine- and vehicle-exposed rats 40 days after the last treatment. Differentially regulated genes were validated by quantitative reverse transcription PCR (qRT-PCR) analysis using independent samples. Gene ontology analysis showed that the majority of upregulated genes had a function in myelination. To assess whether genes involved in myelination were also affected by early-life exposure to fluoxetine, we performed qPCR analysis on the genes involved in myelination in a group of rats neonatally exposed to fluoxetine or vehicle. Finally, we performed correlational analysis between anxiety-like behavior and messenger RNA (mRNA) expression.

## Materials and methods

### Animals

**Figure 1:**
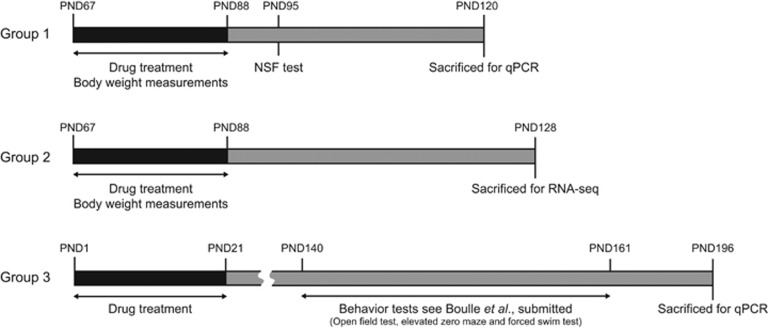
Schematic representation of experimental timeline. Male Wistar rats, group 1 (*n*=12 per treatment) and 2 (*n*=4 per treatment), were treated with fluoxetine or vehicle from postnatal day (PND) 67 to 88. During the treatment period, body weight was measured every day. In group 1, anxiety-like behavior was tested on PND 95 using the novelty-suppressed feeding test (NSFT). Groups 1 and 2 were killed by decapitation on PND 120 and PND 128, respectively and used for mRNA expression analysis using hippocampal tissue. Group 3 (fluoxetine *n*=6, vehicle *n*=7) was used to investigate the effect of chronic fluoxetine exposure on hippocampal mRNA expression in Sprague Dawley rats. For neonatal exposure, dams were treated during the postpartum period from PND 1 to 21. At PND 21, pups were weaned and group-housed for further examination (two rats per cage). Anxiety- and depression-related behavior was analyzed from PND 140 onwards (in the order as written in the figure) and the rats were killed by decapitation at PND 196. mRNA, messenger RNA; qPCR, quantitative PCR.

Male Wistar rats (*Rattus norvegicus*) were obtained from Charles River (Cologne, Germany) and used for experiments after at least 7 days of acclimatization. All the animals were housed per two in standard Macrolon type 3 cages in temperature-controlled rooms (21 °C±1 °C) under a standard 12/12-h day/night cycle (lights on at 0700 h) with food (Sniff, long-cut pellet, Bio Services, Uden, The Netherlands) and water available *ad libitum*. Environmental conditions (for example, housing, light conditions (80 lux), noise level) were carefully controlled as these conditions can strongly influence stress levels in rats.^67, 68^ Three groups of animals were used in this study. In each group, the rats were randomly assigned to a treatment. The investigator was not blinded to the group allocations when performing the experiments, because effects of fluoxetine on the body weight and behavior were clearly visible. Group 1 was treated at adulthood with fluoxetine or vehicle (*n*=12 per treatment), used for body weight measurements during treatment, tested in the NSFT and decapitated to collect hippocampal tissue for qRT-PCR validation; group 2 was treated at adulthood with fluoxetine or vehicle (*n*=4 per treatment) and used for RNA-seq experiments. Finally, group 3 consisted of adult female Sprague Dawley rats neonatally exposed to fluoxetine or vehicle (PND 1 to 21) via osmotic minipumps implanted in the mothers. Their hippocampal tissue was obtained from Maastricht University (fluoxetine *n*=6, vehicle *n*=7) and used for qPCR analysis. Figure 1 provides a schematic representation of the experimental timeline for each group. For behavior experiments, 12 animals per group were used, because this is the minimum required to achieve sufficient statistical power to establish significant differences (*α*=0.05 and *β*=0.20). For genome-wide gene expression analysis, we used two biological replicates. All the experiments were carried out according to the guidelines for the Care and Use of Mammals in Neuroscience and Behavioral Research (National Research Council 2003), the principles of laboratory animal care, as well as the Dutch law concerning animal welfare.

### Drug treatment

Rats from group 1 and 2 received fluoxetine (12 mg kg^−1^ per day, as used by Olivier *et al.*^42^) or vehicle by oral gavage from PND 67 to 88 in a volume of 5 ml kg^−1^. Fluoxetine was purchased from the Pharmacy of the Radboud University Nijmegen Medical Centre, The Netherlands and dissolved in distilled water. As a vehicle, 1% methylcellulose (Genfarma, Maarssen, The Netherlands) was used, which was the constituent of the fluoxetine pills. Body weight was monitored daily throughout the treatment. Rats from group 3 received fluoxetine via the milk of the dams. Minipumps were implanted subcutaneously in the dorsal region of the dams on PND 1 and filled with either fluoxetine–HCl (Fagron, Waregem, Belgium) dissolved in vehicle (50% propylenediol in saline; 5 mg kg^−1^ per day), or with vehicle, as previously described.^69^

### Novelty-suppressed feeding test

The NSFT was performed as described before.^42^ In short, after food deprivation, male rats (PND 95) of group 1 were placed in one corner of an open arena (50 × 50 cm) containing clean wood chip bedding at the center of which was a filter paper containing a food pellet. The latency (s) to start an eating episode was recorded (maximum time was 900 s). After each rat, the arena was cleaned with ethanol (70%) and dried thoroughly to prevent transmission of olfactory cues.

### Transcriptome sequencing

Rats within group 2 were killed at PND 128, brains were removed and stored at −80 °C. The hippocampus was dissected by punching from 300-micron frozen brain slices, and tissue from two rats was pooled for total RNA isolation and cDNA synthesis. DNA samples were prepared for RNA-seq by end repair, adaptor ligation, size selection and amplification. After quality control of DNA libraries, the samples were sequenced (36 bp, single read) with the Illumina Genome Analyzer IIx platform. Sequences were aligned to the rat rn4 reference genome^70^ and further analyzed using Genomatix software (www.genomatix.de). DAVID (Database for Annotation, Visualization and Integrated Discovery; http://david.abcc.ncifcrf.gov/) was used for gene ontology (GO) analysis. RNA-seq validation was performed by qRT-PCR analysis in an independent group of rats (group 1). See Supplementary Information for detailed information about the transcriptome analysis and primer sequences (Supplementary Table S1).

### Quantitative reverse transcription PCR

Hippocampal tissue of rats within group 3 was crunched in liquid nitrogen. RNA was isolated (RNeasy lipid tissue kit; QIAGEN, Venlo, The Netherlands) and cDNA was synthesized using iScript cDNA Synthesis Kit (Bio-Rad, Veenendaal, The Netherlands) according to the manufacturer's protocols. The qPCR reactions were performed in 7500 Fast Real Time PCR System (Applied Biosystems, Foster City, CA, USA) using the SYBR Green fluorescence quantification system (GoTaq qPCR Master Mix, Promega, Leiden, The Netherlands). See Supplementary Information for detailed information about the qRT-PCR method.

### Statistical analysis

Statistical analysis of the data was carried out using the IBM Statistical Package for the Social Sciences (SPSS) version 20.0 (IBM, Armonk, NY, USA). The Shapiro–Wilk test was used to check for normal distributions. Independent samples *t*-tests were used for normally distributed data (corrected *P*-value was used when equal variance was not assumed) and Mann–Whitney *U*-tests for non-normal distributions. Body weight was analyzed by repeated measures analysis of variance and further analyzed per day using independent samples *t*-tests. Spearman correlations were performed for the correlational analysis between behavior tests and mRNA expression. Outliers (data points further than three interquartile ranges from the nearer edge of the box plot) were excluded from the analysis. Independent samples *t*-tests and correlations were performed two-sided. No adjustments for multiple comparison was applied for the RNA-seq. We performed qPCR validations afterwards to validate the RNA-seq results. The level of statistical significance was set at *P*<0.05 in all the tests.

## Results

### Body weight and anxiety-like behavior in response to adult fluoxetine exposure

**Figure 2:**
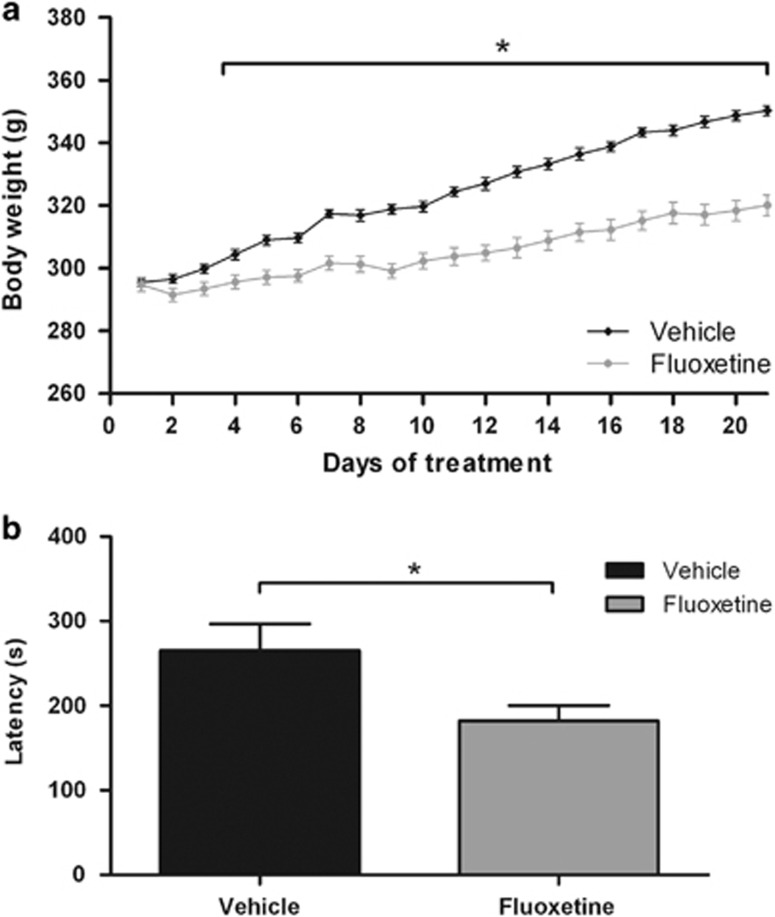
Fluoxetine in adult exposed Wistar rats reduces body weight and latency to start eating in a novel environment. (**a**) Body weight in fluoxetine- and vehicle-treated (postnatal day (PND) 67 to 88) adult male rats (*n*=12/group) measured during the treatment period. Data are presented as mean±s.e.m. of body weight (g). (**b**) Latency to start eating in a novel environment tested in fluoxetine- and vehicle-treated adult male rats on PND 95. Data are presented as mean+s.e.m. of latency (s) to start eating. *P<0.05.

Body weight was measured daily during the treatment period. All the rats received a daily oral administration of fluoxetine or vehicle from PND 67 to 88 (Figure 1). As shown in Figure 2a, starting weight in group 1 was not different between fluoxetine and vehicle groups (*t*_(1,22)_=0.26; *P*=0.796). Repeated measures analysis of variance revealed that fluoxetine significantly reduced adult body weight gain (*F*_(1,22)_=43.37; *P*<0.01). Independent samples *t*-tests indicated that the reduction in body weight gain was significant (*P*<0.05) from day 4 of the treatment and further on. Vehicle-exposed rats grew on average from 295.5 g on the first day of treatment to 350.3 g on the last day of treatment, while fluoxetine-exposed rats grew on average from 294.8 g on the first day of treatment to 320.2 g on the last day of treatment (see Supplementary Table S2 for all body weight values). Similar results were obtained for group 2 (data not shown). Anxiety-like behavior was measured 1 week after treatment using the NSFT. We found that adult fluoxetine-exposed rats exhibited a shorter latency to start eating compared with vehicle-exposed animals (*t*_(1,19)_=2.32; *P*<0.05; Figure 2b, Supplementary Table S2). Both decreased weight gain during chronic fluoxetine exposure^71^ and a shorter latency to start eating in the NSFT after chronic fluoxetine exposure^64, 65, 66, 72^ are consistent with previous findings in stressed and unstressed rats.

### Long-term consequences of adult chronic fluoxetine exposure on genome-wide mRNA expression patterns in the hippocampus

**Figure 3:**
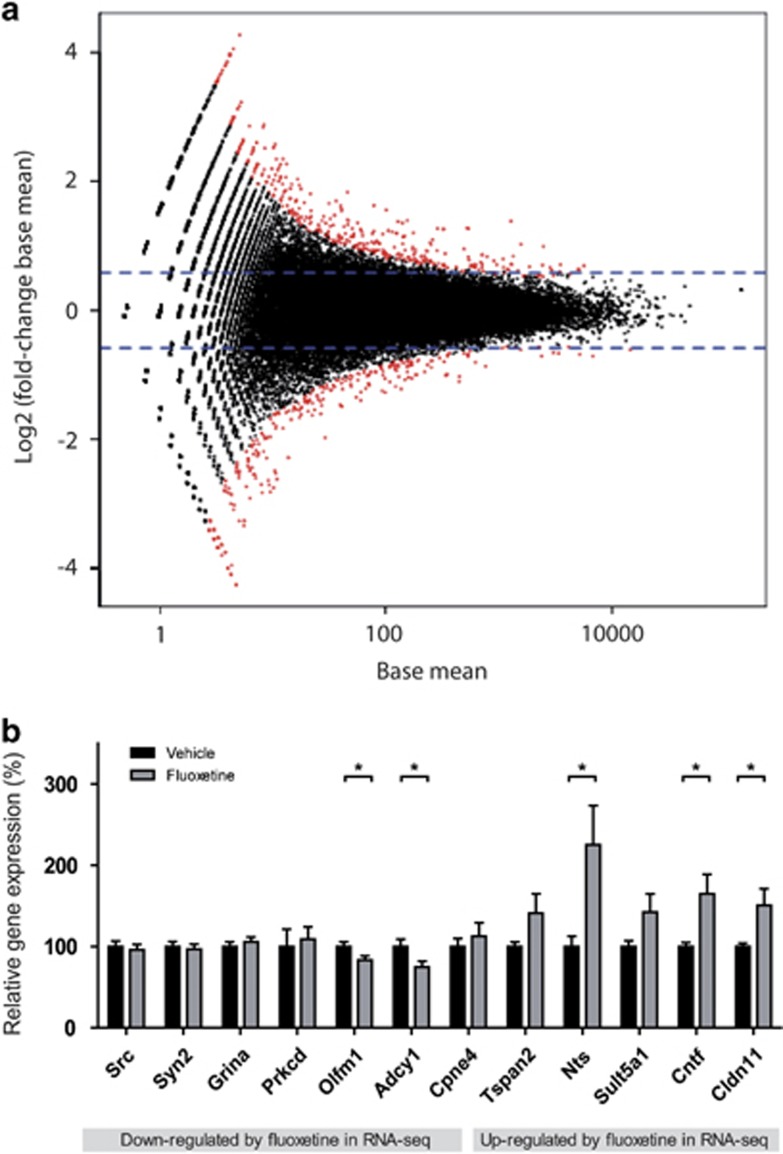
Gene expression in adult fluoxetine-exposed male Wistar rats. (**a**) RNA-seq analysis was performed using hippocampal tissue of fluoxetine- and vehicle-exposed rats, two rats pooled per sample, two samples per treatment group. Fold change scatter plot showing fold change in expression (base mean) in fluoxetine-treated versus vehicle-treated (y axis) against expression level (x axis). Differentially regulated genes are genes with fold change threshold >1.5 (log2 fold change >0.58, blue dashed line) and *P*-value <0.05 (colored in red). Red dots above the upper blue dashed line are upregulated genes (258 genes) and red dots below the lower blue dashed line are downregulated genes (218 genes). (**b**) Validation of RNA-seq results by quantitative RT-PCR (qRT-PCR) analysis in independent biological replicates. Quantitative RT-PCR was performed on hippocampal RNA of adult fluoxetine- and vehicle-treated (postnatal (PND) day 67 to 88) rats (*n*=12 per treatment). On the basis of RNA-seq data, seven genes downregulated (left side in figure) and five genes upregulated (right side in figure) by fluoxetine exposure were selected for qRT-PCR validation. Data are normalized for *Ywhaz* and *Hprt* mRNA levels and are presented as mean+s.e.m. of relative gene expression (% of vehicle group). *P<0.05 indicate genes differentially expressed in qRT-PCR. mRNA, messenger RNA.

To investigate which genetic pathways have a role in the long-term effects of chronic SSRI exposure, RNA-seq analysis was performed using hippocampal tissue of fluoxetine- and vehicle-exposed rats (two rats pooled per sample, two samples per treatment group). Genes with a fold change >1.5-fold and a *P*-value <0.05 were considered as differentially regulated genes. Analysis of the samples resulted in 258 genes that were significantly upregulated and 218 genes that were significantly downregulated by fluoxetine treatment (Figure 3a, Supplementary Table S3). Some genes show overlap with a study in mice chronically treated with fluoxetine (see green marked genes in Supplementary Table S3).^73^

To functionally categorize the differentially expressed genes, GO analysis was performed. The most significantly enriched GO terms in the list of upregulated genes induced by adult fluoxetine treatment are all involved in glia cell development and myelination (Table 1). Examples of upregulated genes involved in glia cell development are *zinc finger protein 488* (*Znf488*), *proteolipid protein 1* (*Plp1*), *ciliary neurotrophic factor* (*Cntf*), *NK6 homeobox 2* (*Nkx6-2*) and *POU class 3 homeobox 1* (*Pou3f1*). For the genes downregulated after adult fluoxetine treatment, the most significantly enriched GO term was ‘response to abiotic (non-living) stimulus'. An underlying and more specific GO term that was also significantly enriched is ‘response to temperature stimulus (an abiotic stimulus)', including genes such as *adrenoceptor beta 2* (*Adrb2*), *nitric oxide synthase 1* (*Nos1*), *caspase 8* (*Casp8*), *transient receptor potential cation channel, subfamily V, member 3* (*Trpv3*), *interleukin 1 beta* (*Il1b*), *chemokine (C-X-C motif) ligand 12* (*Cxcl12*) and *protein kinase C, delta* (*Prkcd*). See Supplementary Table S4 for a complete list of significantly enriched GO terms, including the genes linked to these terms.

Validation of the adult RNA-seq data was performed by qRT-PCR analysis in independent biological replicates (*n*=11–12 per treatment). For validation, we selected 12 differentially regulated genes (five up- and seven downregulated) on the basis of *P*-value (*P*<0.05), fold change (>1.5) and expression profile using the WIG files. Five genes, *olfactomedin 1* (*Olfm1, U=*31.00, *P*<0.05; downregulated), *adenylate cyclase 1* (*Adcy1, U*=33.00, *P*<0.05; downregulated), *neurotensin* (*Nts,U*=25.00, *P*<0.05; upregulated)*, Cntf* (*U*=26.00; *P*<0.05; upregulated) and *claudin 11* (*Cldn11, U*=25.00, *P*<0.05; upregulated), showed a significant change in mRNA expression in the same direction as in the RNA-seq data (Figure 3b). Interestingly, three out of the five significantly upregulated genes in RNA-seq were significantly upregulated in qRT-PCR analysis and the other two genes also showed a change in the right direction, that is, upregulation in the fluoxetine-exposed rats. However, the majority of the genes downregulated in the RNA-seq were not changed in the qRT-PCR analyses, indicating that the upregulated genes were more consistent among independent experiments. Of the upregulated genes, *Cntf, Cldn11* and *Tspan2* (*P=0.17*) are involved in myelination,^74, 75, 76^ indicating that myelination is one mechanism involved in the long-term effects of SSRI exposure.

### Long-term consequences of neonatal chronic fluoxetine exposure on hippocampal mRNA expression

**Figure 4:**
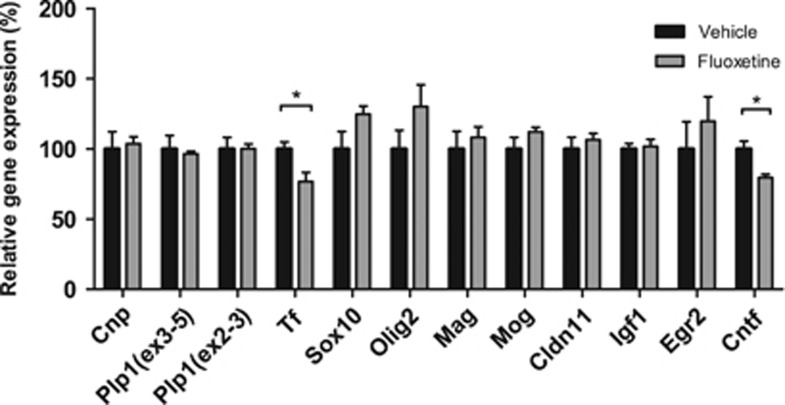
Hippocampal mRNA expression levels in neonatally fluoxetine-exposed female Sprague Dawley rats. Quantitative RT-PCR analysis was performed on hippocampal RNA of adult rats (fluoxetine *n*=6, vehicle *n*=7) neonatally exposed (postnatal day (PND) 1 to 21) to fluoxetine or vehicle. Data are normalized for *Ywhaz* and *Hprt* mRNA levels and are presented as mean+s.e.m. of relative gene expression (% of vehicle group). *P<0.05. mRNA, messenger RNA.

As GO analysis showed that upregulated genes are enriched for genes involved in myelination, we investigated whether myelin-linked genes were also affected in adult rats neonatally exposed to fluoxetine. We had access to the hippocampal tissue of adult rats exposed to fluoxetine or vehicle from PND 1 to 21 and performed qRT-PCR analysis for several genes involved in myelination (based on Aston *et al.*^77^). Expression of *Cntf,* a gene also detected and validated in the RNA-seq experiment, was significantly reduced in response to neonatal fluoxetine exposure compared with vehicle (*U*=6.00, *P*<0.05). In addition, a significant reduction after fluoxetine exposure was found for *transferrin* (*Tf, U*=4.00, *P*<0.05; Figure 4). Consistent with the long-term effects on gene expression after chronic fluoxetine treatment during adulthood, these data show that genes associated with myelination are also involved in the long-term effects of neonatal SSRI exposure, but in the opposite direction.

### Correlation between behavior and expression of myelination-related genes

To investigate whether the anxiolytic effect of chronic SSRI exposure (see section ‘Body weight and anxiety-like behavior in response to adult fluoxetine exposure') is related to the altered expression of myelination-related genes, we performed a correlational analysis. Group 1 was used for both the NSFT and qPCR validations, which enables correlational analysis between latency to start eating and mRNA expression (Supplementary Figure S1). Interestingly, we found a negative correlation (*r*_(18)_=−0.529, *P*<0.05) between *Cldn11* mRNA expression and the latency to start eating in the NSFT. In addition, we found a trend for a negative correlation between *Tspan2* mRNA expression and latency to start eating (*r*_(18)_=−0.412, *P*<0.1).

We also performed a correlational analysis using data (anxiety-like behavior in an open-field test (OFT, results see Boulle and colleagues^78^) and expression analysis of myelination-related genes) derived from the neonatally fluoxetine-exposed rats (group 3). In the OFT, time spent in the corner (OFC) and time spent in the center of the open field were measured, in which OFC is a measure for anxiety-like behavior and time spent in the center of the open field is a measure for anxiolytic-like behavior. We found that OFC correlated negatively with mRNA expression of *Cldn11* (*r*_(11)_=−0.736, *P*<0.05), *Cnp* (*r*_(11)_=−0.682, *P*<0.05), *Plp1* (ex3–5) (*r*_(11)_=−0.827, *P*<0.05) and *Plp1* (ex2–3) (*r*_(11)_=−0.800, *P*<0.05). In addition, *Mag* mRNA expression showed a trend for a negative correlation with OFC (*r*_(11)_=−0.555, *P*<0.1). Finally, a trend for a positive correlation with time spent in the center of the open field was found for mRNA expression of *Cldn11* (*r*_(11)_=0.582, *P*<0.1), *Plp1* (ex3–5) (*r*_(11)_=0.527, *P*<0.1) and *Plp1* (ex2–3) (*r*_(11)_=0.536, *P*<0.1) and *Mog* (*r*_(11)_=0.573, *P*<0.1). See Supplementary Figure S1–S3 for a complete overview of the correlation data.

Taken together, these data indicate that a higher expression of myelination-related genes is linked to anxiolytic-like behavior in both the NSFT in adult fluoxetine-exposed rats and the OFT in the neonatally fluoxetine-exposed rats.

## Discussion

In this study, we demonstrate, using a genome-wide approach, that 40 days after chronic fluoxetine treatment in adult rats mRNA levels of myelination-related genes were significantly upregulated in the hippocampus. Interestingly, in an independent group of rats, we observed that chronic neonatal fluoxetine exposure downregulated myelination-related genes. We specifically observed that the myelination-related *Cntf* gene was upregulated in adult fluoxetine-exposed rats and downregulated in neonatally fluoxetine-exposed rats. In addition, we observed a negative correlation between expression of myelination-related genes and anxiety-like behavior in both the adult and neonatally fluoxetine-exposed rats. These data suggest that chronic SSRI exposure exerts its long-term effects, among others, by affecting myelination processes.

There are other studies in rodents showing genome-wide gene expression differences after adult fluoxetine treatment, but so far they all focused on short-term effects by investigating gene expression 1 day after the last fluoxetine administration.^73, 79, 80, 81^ The present finding that myelination-related genes were affected more than 40 days after chronic SSRI exposure, both in early life and adulthood, is important given that it elucidates the neurobiological mechanisms contributing to the development of (early-life exposure) and recovery from (adult exposure) psychiatric disorders. Interestingly, there is overlap in differentially regulated genes between studies focusing on short-term effects and our study about long-term effects. For instance, Samuels *et al.*^73^ performed a microarray study using dentate gyrus tissue from 24 h after treatment cessation of adult mice chronically treated with fluoxetine and identified eight upregulated and 20 downregulated genes that overlap with our findings (see green marked genes in Supplementary Table S3). Genes affected in both short- and long-term studies might have a crucial role in inducing and maintaining the antidepressant state. It is not likely that effects of fluoxetine withdrawal are seen in our expression data, because these effects occur shortly after withdrawal and will not last for 40 days.

RNA-seq validation by qPCR showed that the upregulated genes were more consistent among independent experiments. We were unable to validate five out of the seven downregulated genes, therefore, we focused on the upregulated genes. The GO analysis of genes upregulated by chronic fluoxetine exposure in adulthood revealed that the majority of these genes have a function in myelination. In addition, we found a correlation between the latency to start eating in the SSRI-sensitive NSFT and gene expression of myelination-related genes (*Cldn11,* and a trend for *Tspan2*), which strengthens our findings. Interestingly, a wide range of psychiatric disorders responsive to SSRI treatment, including depression, bipolar affective disorder, obsessive compulsive disorder, post-traumatic stress disorder and autism spectrum disorder have been associated with defects in white matter, which consists mainly of myelinated axons.^82, 83^ A first link between mood disorders and myelin was shown by Aston *et al.*^77^ They studied gene expression in the temporal cortex of major depressive disorder patients and found a decreased expression of genes encoding structural components of myelin (for example, *2',3'-cyclic nucleotide 3' phosphodiesterase* (*CNP*)*,* *myelin-associated glycoprotein* (*MAG*)*,* *myelin oligodendrocyte glycoprotein* (*MOG*)*, PLP1*) and genes involved in myelin formation (for example, *TF, SRY* (*sex determining region Y*)*-box 10* (*SOX10*)). We showed in our RNA-seq experiment that the SSRI fluoxetine increases the expression of genes linked to myelination in the hippocampus. Interestingly, we did not find the same genes as Aston *et al.* found in the temporal cortex (gene expression might be brain region dependent), but we did find genes (*Cntf, Cldn11*) influencing the same process. Genes interacting with each other (*SOX10* and *Cntf*^84^) and genes with similar functions regarding myelination (*PLP1* and *Cldn11* (ref. 85) are found in the study by Aston *et al.* and our RNA-seq experiment. Moreover, in obsessive compulsive disorder patients, abnormalities of myelin integrity have been found that were partially reversed by SSRI treatment.^53^ Taken together, these findings suggest that myelination is dysregulated in several psychiatric disorders and can be regulated by antidepressants, like fluoxetine.

In hippocampal tissue of neonatally fluoxetine-exposed rats, we found that *Cntf* was downregulated, which directly opposed the finding that adult fluoxetine exposure upregulated this very same gene. Thus, the same gene, *Cntf*, was affected in the opposite direction by chronic fluoxetine exposure in early life and adulthood. This is consistent with the growing amount of experimental evidence that early-life SSRI exposure leads to ‘paradoxical' autism-, anxiety- and depression-like symptoms in later life.^1, 42, 43, 44^ In agreement, our neonatally fluoxetine-exposed rats showed increased depression-like behavior (forced-swim test) at adulthood compared with the neonatally vehicle-exposed rats.^78^ Furthermore, Boulle *et al.*,^78^ showed that neonatal fluoxetine exposure decreased *Bdnf* IV expression in hippocampus, whereas others observed increased *Bdnf* expression in hippocampus of adult fluoxetine-exposed rats 24 h after treatment cessation.^86^ *Cldn11* expression, which was upregulated in the adult fluoxetine-exposed group, was not found to be regulated in the opposite direction (downregulated) in the neonatally fluoxetine-exposed group.

The decrease in expression of the two myelin-linked genes after early-life fluoxetine exposure is in line with the findings of Simpson *et al.*^40^ They showed that early-life SSRI exposure (citalopram) disturbs myelin sheath formation and decreases interhemispheric connectivity by 50%. In addition, high levels of serotonin can lead to aberrant oligodendrocyte development and myelination deficits *in vitro.*^87^ Our results of the qRT-PCR in hippocampus tissue of early-life fluoxetine-exposed rats suggest that gene expression of myelination-related genes was also affected by SSRIs. Notably, our adult and neonatally fluoxetine-exposed groups differed in fluoxetine dose, strain and gender, making it possible that the opposite finding was driven by these factors rather than neonatal versus adult fluoxetine exposure. However, our finding that expression of the myelination-related *Cldn11* gene and anxiety correlated negatively in both the adult and neonatally fluoxetine-exposed rats does not support this. Given that changes in myelination have been reported by others after both neonatal^40^ and adult^53^ SSRI exposure, it is more likely that our findings are the result of fluoxetine exposure at different ages.

The *Cntf* gene, coding for ciliary neurotrophic factor, is the only gene differentially regulated in all our experimental groups. CNTF is a neurotrophic factor produced by astrocytes, which supports the proliferation^88^ and survival^89, 90, 91^ of oligodendrocyte precursors and regulates myelination.^74^ Studies have shown that CNTF can mediate stroke-induced adult central nervous system neurogenesis^92^ and that CNTF injection can increase remyelination in cuprizone-induced multiple sclerosis mice,^93^ supporting the role of CNTF as a neurotrophic factor and as a myelin regulator. In the hippocampus, *Cntf* is strongest expressed in the dentate gyrus and CA1 regions.^94^ The dentate gyrus is important for adult neurogenesis and therefore *Cntf* expression in this region fits well with its role in neurogenesis. Studies have shown that CNTF is essential for the formation and/or maintenance of the neurogenic subgranular zone in the adult dentate gyrus.^95^ How fluoxetine targets myelination-related genes is still unclear. On the basis of literature, we propose a potential pathway, but this is highly speculative (see Supplementary Figure S4). In short, fluoxetine stimulates the 5-HT2B receptor on astrocytes resulting in activation of its downstream signaling cascades,^96^ which potentially can lead to release of CNTF. The released CNTF can activate astrocytes and these astrocytes then release an astrocyte-specific factor (>30 kDa), which promotes proliferation and survival of oligodendrocyte precursor cells^97^ and maturation of oligodendrocytes.^90, 98^ Of further interest, *Cntf−/−* mice display increased anxiety- and depression-like behavior.^99^ These findings are in line with the reduced *Cntf* expression that we found in the group of rats exposed to fluoxetine at early life, which also showed increased depression-like behavior.^78^ *Cldn11* expression is upregulated by adult chronic fluoxetine exposure and showed a negative correlation with anxiety-like behavior in the NSFT. In the neonatally fluoxetine-exposed rats, *Cldn11* expression also showed a negative correlation with anxiety-like behavior (OFC) in the OFT, despite the absence of significant differences between the treatment groups in the OFT and the expression analysis. *Cldn11* codes for Claudin-11, which is a major component of myelin and forms tight junctions within myelin sheaths.^100^ Downregulation of *Cldn11* has been found in bipolar affective disorder patients.^101^ Also *Plp1* and *Cnp* showed a negative correlation with anxiety-like behavior (OFC) in the OFT. Taken together, the correlations indicate that a higher expression of myelination-related genes results in anxiolytic-like behavior.

In this study, we found that fluoxetine can cause long-term changes in the expression of myelination-related genes. However, a potential limitation of the present study is that we used a homogenate of hippocampus cells and there are different cell types in the hippocampus tissue. Selecting a specific cell type using fluorescence-activated cell sorting might give more insights in the gene expression per cell type, although it is notable that mRNA levels correlated with behavior. Another limitation of this study is that the fluoxetine dose differs between the prenatally (5 mg kg^−1^ per day) and adult (12 mg kg^−1^ per day) exposed groups. However, studies have shown that exposure to higher doses of fluoxetine early in life (10–20 mg kg^−1^ per day) affects anxiety-like behavior (for example, OFT) in the same way as seen for 5 mg kg^−1^.^102, 103^ Furthermore, studies using a lower dose of fluoxetine in adulthood (5 mg kg^−1^ per day) showed a similar effect on anxiety-like behavior in the NSFT as shown in this study for 12 mg kg^−1^ per day.^65^ In the future, it is relevant to explore whether fluoxetine exposure will give similar results in models for psychiatric disorders responsive to SSRIs. Given that the effect of SSRIs in the NSFT is the same in healthy^64, 65^ and stressed^66, 72^ animals it is likely that also gene expression patterns will be similar. Finally, we measured gene expression, and it remains to be established whether our findings translate to changes in protein levels and myelination. As a next step in biology, evidence of changes in myelination will further support our findings. As such, it has already been demonstrated that SSRI treatment can have consequences for myelination.^40, 53, 104^

In conclusion, we show that adult and neonatal chronic fluoxetine exposure cause long-term changes in hippocampal expression of ciliary neurotrophic factor and other genes linked to myelination, a process that shapes brain connectivity and could contribute to the remediation of symptoms of psychiatric disorders, like anxiety.

## Figures and Tables

**Table 1:** Significantly enriched GO terms (biological process) affected by adult fluoxetine treatment

*Factor*	*DAVID ID*	*GO term*	*No. of genes*	P*-value*	*Fold enrichment*
Upregulated by fluoxetine	GO:0021782	Glial cell development	5	4.4E−4	13.7
	GO:0010001	Glial cell differentiation	6	7.1E−4	8.4
	GO:0042063	Gliogenesis	6	2.0E−3	6.7
	GO:0042391	Regulation of membrane potential	8	2.5E−3	4.3
	GO:0008366	Axon ensheathment	5	2.6E−3	8.6
	GO:0007272	Ensheathment of neurons	5	2.6E−3	8.6
	GO:0001508	Regulation of action potential	6	3.1E−3	6.0
	GO:0033205	Cytokinesis during cell cycle	3	3.9E−3	30.8
Downregulated by fluoxetine	GO:0009628	Response to abiotic stimulus	15	8.2E−5	3.5
	GO:0051931	Regulation of sensory perception	4	7.1E−4	22.1
	GO:0051930	Regulation of sensory perception of pain	4	7.1E−4	22.1
	GO:0009266	Response to temperature stimulus	7	7.7E−4	6.4
	GO:0035239	Tube morphogenesis	8	1.3E−3	4.8
	GO:0043044	ATP-dependent chromatin remodeling	3	2.3E−3	40.2
	GO:0044236	Multicellular organismal metabolic process	4	2.8E−3	13.9
	GO:0060562	Epithelial tube morphogenesis	6	4.0E−3	5.7

Abbreviations: DAVID, Database for Annotation, Visualization and Integrated Discovery; GO, gene ontology.
