# Supplementary material for: Long-term consequences of chronic fluoxetine exposure on the expression of myelination-related genes in the rat hippocampus
Source: Transl Psychiatry. 2015 Sep 22;5(9):e642–. doi: 10.1038/tp.2015.145 (PMC5068807; doi:10.1038/tp.2015.145)
Supplement: Supplementary Information [file tp2015145x1.doc]

**Supplementary information**

1. Supplementary material and methods
2. Supplementary references
3. Table S1: Primers used for quantitative PCR
4. Table S2: Body weight and novelty suppressed feeding test measurements
5. Figure S1: Correlations between novelty suppressed feeding test and expression of myelination-related genes
6. Figure S2: Correlations between time spent in the corner of the open field and expression of myelination-related genes
7. Figure S3: Correlations between time spent in the center of the open field and expression of myelination-related genes
8. Figure S4: Potential pathway for fluoxetine affecting myelination-related genes

**Supplementary materials and methods**

**RNA extraction and Double-Stranded cDNA Synthesis**

Animals of group 2 were sacrificed at PND 128, brains were removed and immediately placed on dry ice and stored at -80°C. Hippocampal tissue was dissected in 8 consecutive slices of 200 µm using a 2 mm punch needle. Tissue from 2 rats was pooled for total RNA isolation with QIAzol (RNeasy lipid tissue kit; QIAGEN, Venlo, The Netherlands) according to the manufacturer's recommendations. Total RNA was subjected to two rounds of poly(A) selection (Oligotex mRNA Mini Kit; QIAGEN), followed by DNaseI treatment (QIAGEN) and fragmentation by hydrolysis (5× fragmentation buffer: 200mM Tris acetate, pH8.2, 500mM potassium acetate and 150mM magnesium acetate) at 94°C for 90 s. Fragmented mRNA was purified (RNeasy MinElute Kit; QIAGEN) and used for cDNA synthesis with 5 μg random hexamers by Superscript III Reverse Transcriptase (Invitrogen Life technologies, Bleiswijk, The Netherlands). Double stranded cDNA synthesis was performed in second strand buffer (Invitrogen) according to the manufacturer's recommendations and purified using the Minelute Reaction Cleanup Kit (QIAGEN) according to the manufacturer's protocol.

**Sequencing**

DNA samples were prepared for RNA-seq by end repair of 20 ng DNA as measured by Qubit dsDNA HS (Invitrogen). Adaptors were ligated to DNA fragments, followed by size selection (~300 bp) and 14 cycles of PCR amplification. Quality control of DNA libraries prepared for sequencing was made by qPCR and by running the products on a Bioanalyzer (Bio-Rad, Veenendaal, The Netherlands). Cluster generation and sequencing (36 bp, single read) was performed with the Illumina Genome Analyzer IIx (GAIIx) platform according to standard Illumina protocols. Samples were sequenced to a depth of approximately 15 million uniquely mapped tags per sample. Sequences were aligned to the rat rn4 reference genome with the Illumina Analysis Pipeline allowing one mismatch. Only the tags aligning to one position on the genome were considered for further analysis. The output data were converted to Browser Extensible Data (BED) files for downstream analysis and Wiggle (WIG) files for viewing.

**Data analysis**

RNA-seq data were analyzed using Genomatix software (www.genomatix.de). The number of sequence reads for each transcript was quantified and additionally a standardized normalized expression (NE) value per transcript was calculated (based on the number of reads located in the exons of the transcript and normalized to the length of the transcript and the density of the data set). NE values of vehicle and fluoxetine samples (group 2) were used to calculate fold change values for each transcript. Genes were identified as differentially expressed if they showed a DESeq [^1^](#_ENREF_1) P-value < 0.05 and fold change (FC) > 1.5 (among two biological replicates for both fluoxetine and vehicle treatment). The Database for Annotation, Visualization and Integrated Discovery (DAVID, http://david.abcc .ncifcrf.gov/) was used for gene ontology (GO) analysis.

**Quantitative Reverse Transcription PCR**

RNA-seq validation was performed by RT-qPCR analysis of selected genes using GoTaq® qPCR Master Mix (Promega Benelux b.v. Leiden, The Netherlands). Primers were designed using Primer3 online software (http://frodo.wi.mit.edu). See table S1 for primer sequences. Complementary DNA (cDNA) was synthesized using 1 μg of total RNA in a reverse transcription reaction using iScript cDNA Synthesis Kit according to manufacturer’s protocol (Bio-Rad). qPCR reactions were performed in a 7500 Fast Real Time PCR System (Applied Biosystems, Foster City, CA, U.S.A) using the SYBR Green fluorescence quantification system (GoTaq® qPCR Master Mix, Promega). Thermal cycling was initiated with incubation at 95°C for 10 min followed by 40 cycles of 95°C for 30 sec and 60°C for 1 min. To normalize the cDNA content of the samples, we used the comparative threshold cycle (CT) method [^2^](#_ENREF_2), which consists of the normalization of the number of target gene copies versus two endogenous reference genes *Ywhaz* and *Hprt1*.

Table S1: Primers used for quantitative PCR

| **Gene symbol** | **Ref-seq number** | **Forward primer** | **Reverse primer** |
| --- | --- | --- | --- |
| *Olfm1* | NM_053573.1 | AGACCTCAGGCTCAAGGTTC | CACCATGGACTTGTACTCACG |
| *Grina* | NM_153308.4 | AGGCTCTTCTGCGTCTTCC | AACTCTTTTCATGGGACATGG |
| *Syn2* | NM_001034020.1 | ATGCGGATGGAACCTACG | GGATGAGCACGAAGTCTGG |
| *Adcy1* | NM_001107239.1 | CTGTGTGGAGATGGGACTTG | CACACGCATGTTCAGGTCTAC |
| *Src* | NM_031977.1 | GGAATCAGAGCGGCTACTTC | TTTCACATTTAGGCCCTTGG |
| *Tspan2* | NM_022589.1 | CAGCTCATTGGAATTGTTGG | AGTTCCGTATTGCACAGCAG |
| *Prkcd* | NM_133307.1 | GCCTTTGTCCTGAATGTGG | CCTTCCTCACCCATCTCATC |
| *Cldn11* | NM_053457.2 | CGCATACAGGAAACCAGATG | CTGGGGTGCTCCTTATTCTG |
| *Cpne4* | NM_001109003.1 | TCATCCTCAAGATGCAATCC | CCACCGTAAACAGCTTTGAG |
| *Nts* | NM_001102381.1 | CTGCTTGTCAGAAGGCTGAG | GATCTGCCTCCAGGACTCTC |
| *Sult5a1* | NM_001201369.1 | CAGAGTCACCCATCTTGGAC | ACCAGAGTCAGGGCAAGTTC |
| *Cntf* | NM_013166.1 | CTTGCCACTGGTACACCATC | TCGTTCAGACCTGACTGCTC |
| *Egr2* | NM_053633.1 | TGCCCATGTAAGTGAAGGTC | TGATCAGATGAACGGAGTGG |
| *Igf1* | NM_001082479.1 | AAAGTCAGCTCGTTCCATCC | TCTTGTTTCCTGCACTTCCTC |
| *Plp1 (ex2-3)* | NM_030990.2 | TCTCCAAAAACTACCAGGACTATG | GGCCCCATAAAGGAAGAAG |
| *Plp1 (ex3-5)* | NM_030990.2 | TTTGGGAAAATGGCTAGGAC | TGCAGATGGACAGAAGGTTG |
| *Olig2* | NM_001100557.1 | TCACAGGAGGAACCGTGTC | TGCTGGAGGAAGATGACTTG |
| *Sox10* | NM_019193.2 | TCTTTGGGGTGGTTGGAG | GCTGCTATCCAGGCTCACTAC |
| *Mag* | NM_017190.4 | AGACAATGGCAATCAGGATG | TTTGTACCTCCAGGAACCTCTAC |
| *Tf* | NM_001013110.1 | GGAAAGTGCAGGCTTCTAGG | CAGAGATGACACCAAGTGTTTG |
| *Mog* | NM_022668.2 | GAGGTTCTCGGATGAAGGAG | CAGGGTTGATCCAGTAGAAGG |
| *Cnp* | NM_012809.2 | GGCAGAAGAATATGCCCAAC | TCACAAAGAGGGCAGAGATG |
| *Hprt1* | NM_012583.2 | GCAGACTTTGCTTTCCTTGG | CGAGAGGTCCTTTTCACCAG |
| *Ywhaz* | NM_013011.3 | TTGAGCAGAAGACGGAAGGT | GAAGCATTGGGGATCAAGAA |

Table S2: Body weight and novelty suppressed feeding test measurements


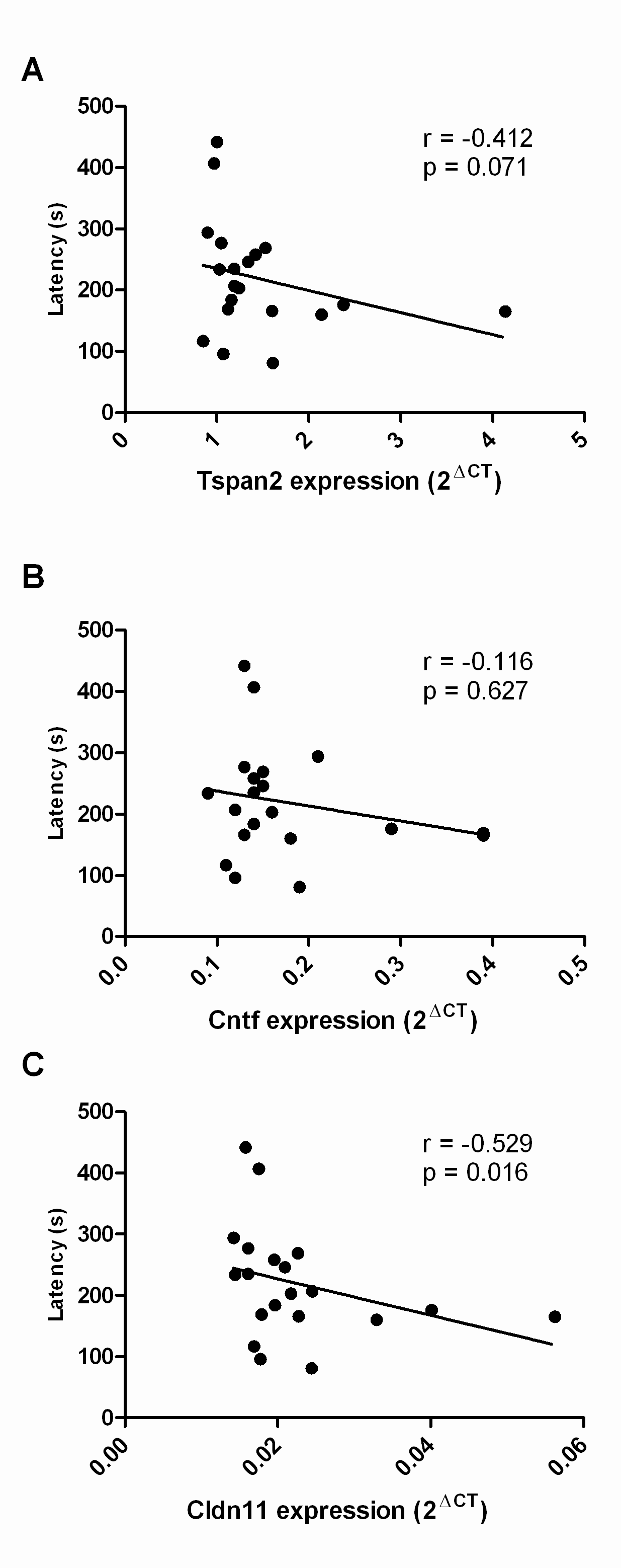


**Fig. S1:** **Correlations between latency to start eating in the novelty suppressed feeding test (NSFT) and expression of myelination-related genes in adult fluoxetine-exposed rats.** Spearman correlations between latency to start eating (s) and expression of myelination-related genes (2^ΔCT^). A significant negative correlation was found for latency to start eating and expression of *Cldn11* (p<0.05) and a trend for a negative correlation was found for latency to start eating and expression of *Tspan2* (p<0.1).


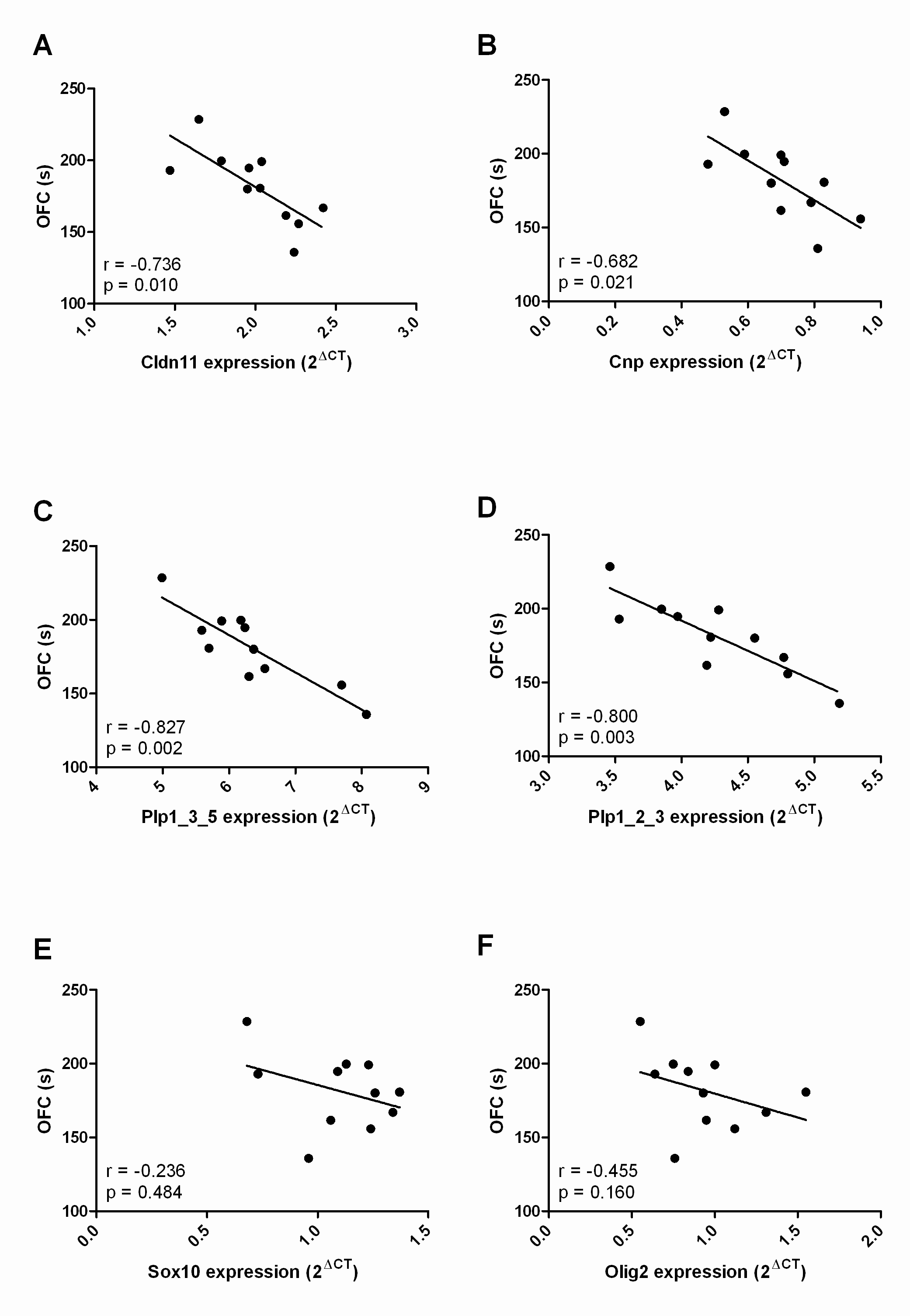


**
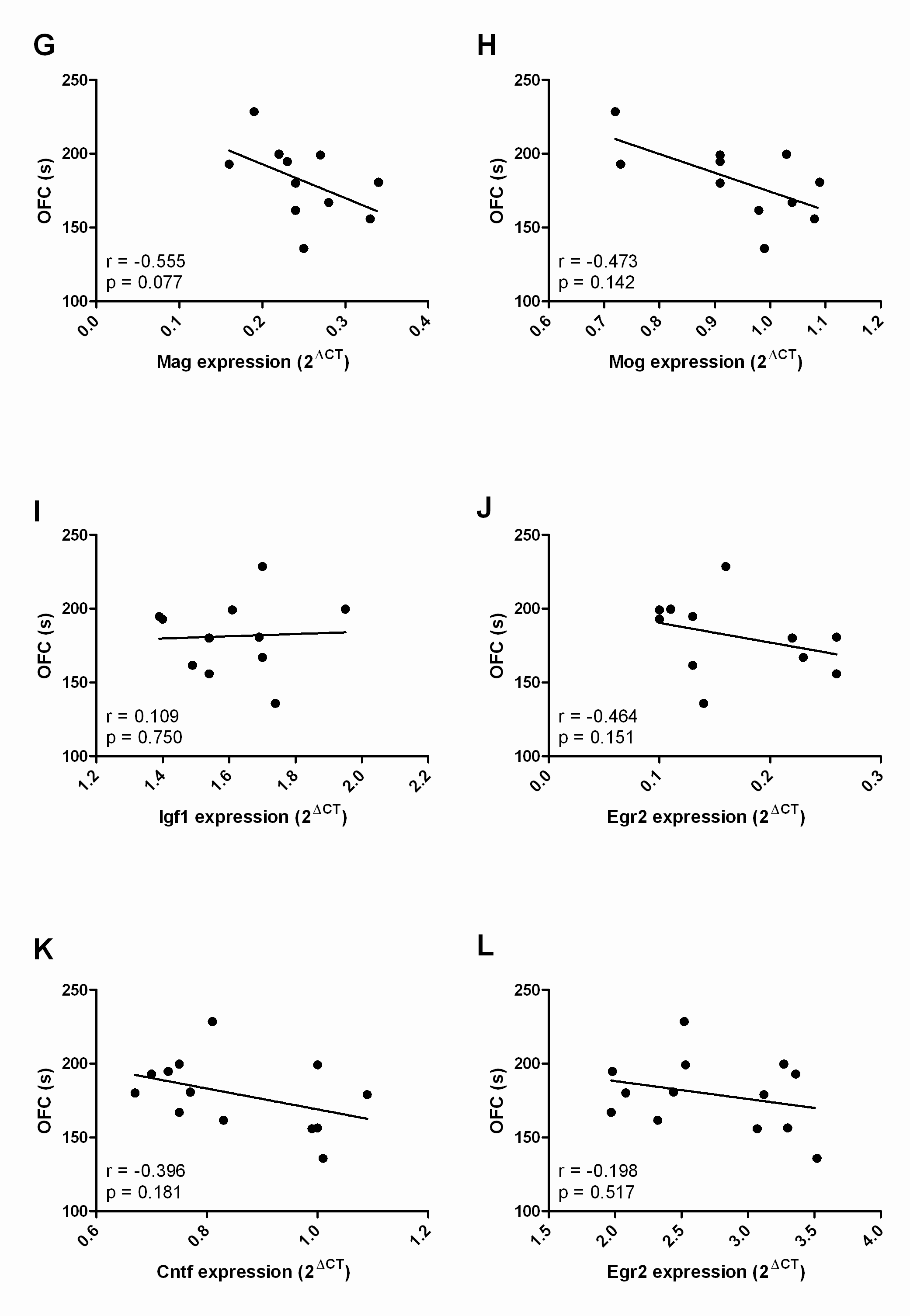
**

**Fig. S2:** **Correlations between time spent in the corner (OFC) and expression of myelination-related genes in neonatally fluoxetine-exposed rats.** Spearman correlations between OFC (s) and expression of myelination-related genes (2^ΔCT^). A significant negative correlation was found for OFC and expression of *Cldn11, Cnp, Plp1_3_5* and *Plp1_2_3* (p<0.05) and a trend for a negative correlation was found for *Mag* (p<0.1).


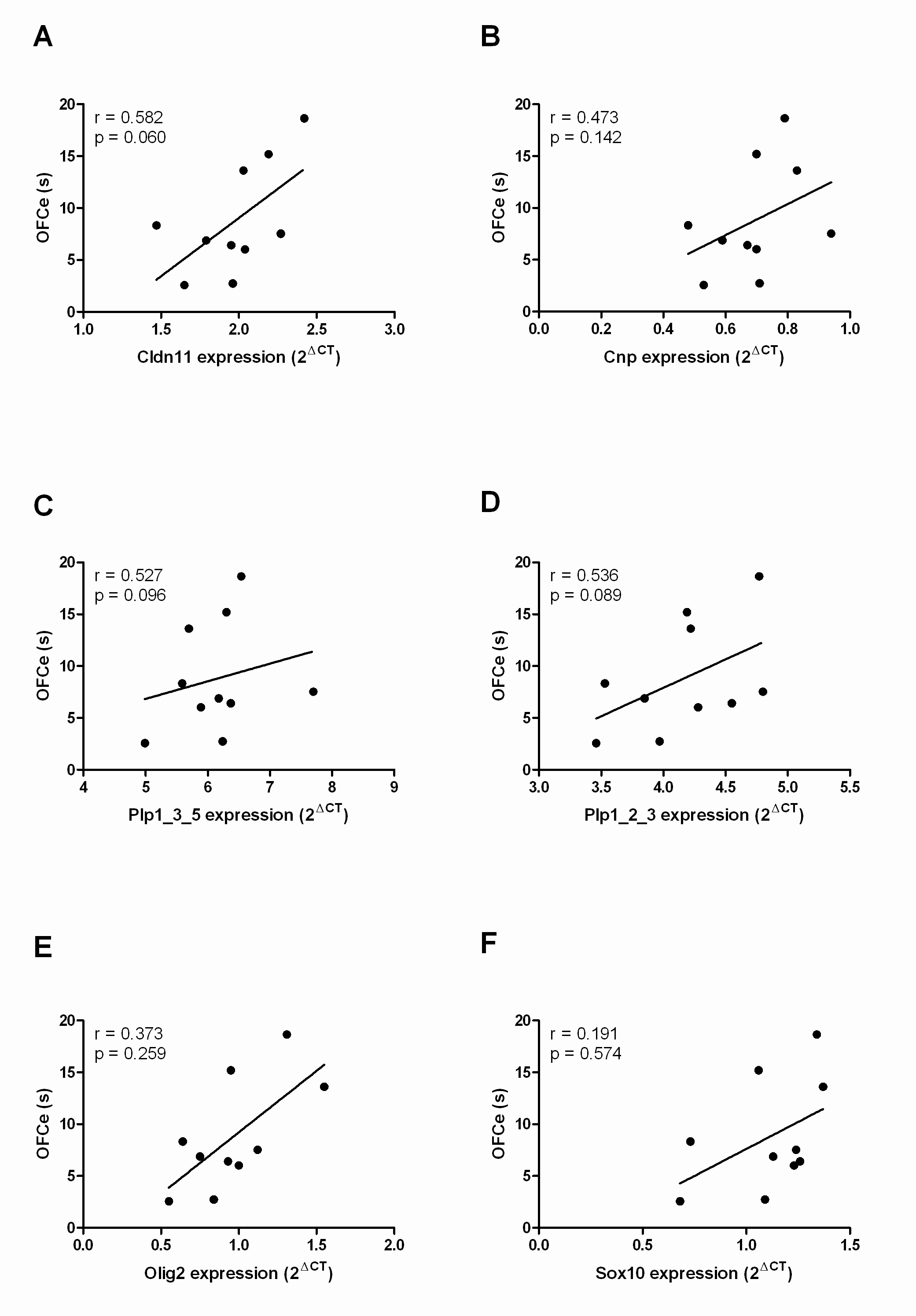


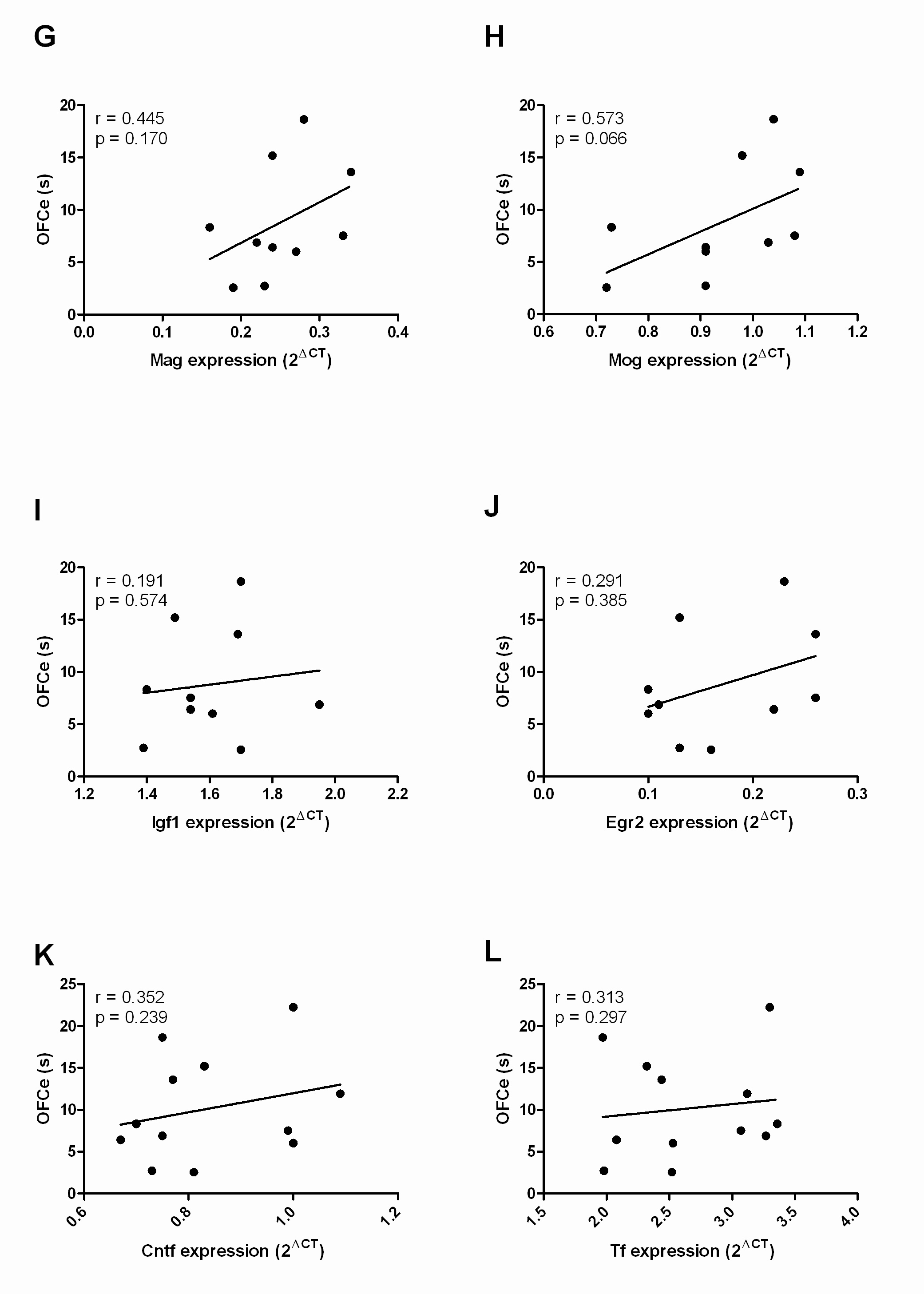


**Fig. S3:** **Correlations between time spent in the center (OFCe) and expression of myelination-related genes in neonatally fluoxetine-exposed rats.** Spearman correlations between OFCe (s) and expression of myelination-related genes (2^ΔCT^). A trend for a positive correlation was found for OFCe and expression of *Cldn11, Plp1_3_5*, *Plp1_2_3* and *Mog* (p<0.1).

**
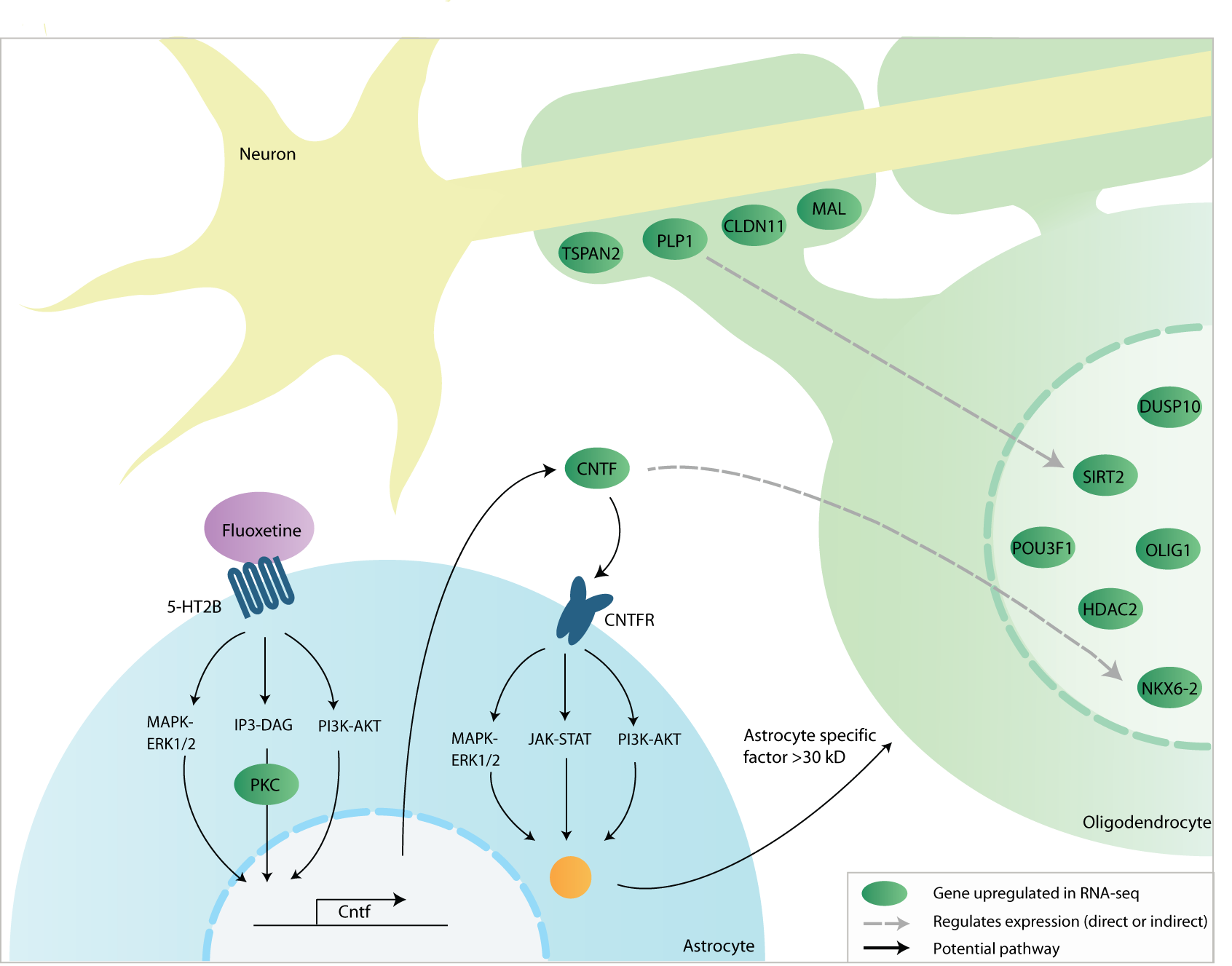
**

**Fig S4: Potential pathway for fluoxetine affecting myelination-related genes**. It has been shown that fluoxetine can stimulate the 5-HT2B receptor on astrocytes, which results in activation of its downstream signaling cascades[^3^](#_ENREF_3). The 5-HT2B receptor is Gq/11 protein coupled and stimulates the diacylglycerol (DAG) and inositol 1,4,5-trisphosphate (IP3) pathway (IP3-DAG). DAG is the physiological activator of protein kinase C (PKC), which in turn can activate the mitogen-activated protein kinase (originally called ERK, extracellular signal-regulated kinase) pathway (MAPK-ERK1/2)[^3^](#_ENREF_3). A third signal transduction pathway activated by fluoxetine-induced stimulation of the 5-HT2B receptor is the PI3K-AKT pathway[^3^](#_ENREF_3)[../../Users/Yvet/Downloads/fnbeh-09-00025.pdf](../../../Users/Yvet/Downloads/fnbeh-09-00025.pdf). Activation of these signal transduction pathways results in transcription factor activation and transcription of neurotrophic factors. MAPK-ERK1/2 activation by fluoxetine results in transcription of glial-derived nerve factor (GDNF)[../../Users/Yvet/Downloads/fnbeh-09-00025.pdf](../../../Users/Yvet/Downloads/fnbeh-09-00025.pdf). In addition, transcription of brain derived neurotrophic factor transcription is induced, but not through the MAPK-ERK1/2 pathway[^4^](#_ENREF_4). Possibly also transcription and release of ciliary neurotrophic factor (CNTF) is triggered by fluoxetine. The released CNTF can bind to the CNTF receptor triggering intracellular signaling through three major signal transduction pathways: JAK- STAT, MAPK-ERK1/2 and PI3K-AKT[^5^](#_ENREF_5). These signal transduction pathways all mediate different responses. Studies have shown that CNTF-activated astrocytes release an astrocyte specific factor (>30 kD), which promotes proliferation and survival of oligodendrocyte precursor cells[^6^](#_ENREF_6). Furthermore, studies have shown that CNTF can induce maturation of oligodendrocytes. An increase in the number of oligodendrocyte precursors and mature oligodendrocytes in our hippocampus tissue might explain the enhanced expression of myelination-related genes. It has been shown that the expression of Nkx6.2 in oligodendrocytes is strongly induced after CNTF treatment (grey dotted line)[^9^](#_ENREF_9) and that Plp1 is required for transport and expression of SIRT2 in myelin (grey dotted line)[^10^](#_ENREF_10).

**Supplementary references**

1. Anders S, Huber W. Differential expression analysis for sequence count data. *Genome biology* 2010; **11**(10)**:** R106.

2. Livak KJ, Schmittgen TD. Analysis of relative gene expression data using real-time quantitative PCR and the 2(-Delta Delta C(T)) Method. *Methods* 2001; **25**(4)**:** 402-408.

3. Hertz L, Rothman DL, Li B, Peng L. Chronic SSRI stimulation of astrocytic 5-HT2B receptors change multiple gene expressions/editings and metabolism of glutamate, glucose and glycogen: a potential paradigm shift. *Frontiers in behavioral neuroscience* 2015; **9:** 25.

4. Mercier G, Lennon AM, Renouf B, Dessouroux A, Ramauge M, Courtin F*, et al*. MAP kinase activation by fluoxetine and its relation to gene expression in cultured rat astrocytes. *J Mol Neurosci* 2004; **24**(2)**:** 207-216.

5. Askvig JM, Watt JA. The MAPK and PI3K pathways mediate CNTF-induced neuronal survival and process outgrowth in hypothalamic organotypic cultures. *Journal of cell communication and signaling* 2015.

6. Albrecht PJ, Enterline JC, Cromer J, Levison SW. CNTF-activated astrocytes release a soluble trophic activity for oligodendrocyte progenitors. *Neurochemical research* 2007; **32**(2)**:** 263-271.

7. Mayer M, Bhakoo K, Noble M. Ciliary neurotrophic factor and leukemia inhibitory factor promote the generation, maturation and survival of oligodendrocytes in vitro. *Development* 1994; **120**(1)**:** 143-153.

8. Stankoff B, Aigrot MS, Noel F, Wattilliaux A, Zalc B, Lubetzki C. Ciliary neurotrophic factor (CNTF) enhances myelin formation: a novel role for CNTF and CNTF-related molecules. *J Neurosci* 2002; **22**(21)**:** 9221-9227.

9. Rivera FJ, Kandasamy M, Couillard-Despres S, Caioni M, Sanchez R, Huber C*, et al*. Oligodendrogenesis of adult neural progenitors: differential effects of ciliary neurotrophic factor and mesenchymal stem cell derived factors. *J Neurochem* 2008; **107**(3)**:** 832-843.

10. Werner HB, Kuhlmann K, Shen S, Uecker M, Schardt A, Dimova K*, et al*. Proteolipid protein is required for transport of sirtuin 2 into CNS myelin. *J Neurosci* 2007; **27**(29)**:** 7717-7730.
